# Dos and don’ts in response priming research

**DOI:** 10.2478/v10053-008-0092-2

**Published:** 2011-12-22

**Authors:** Filipp Schmidt, Anke Haberkamp, Thomas Schmidt

**Affiliations:** Faculty of Social Sciences, Psychology I, University of Kaiserslautern, Germany

**Keywords:** response priming, unconscious perception, research methods

## Abstract

Response priming is a well-understood but sparsely employed paradigm in cognitive science. The method is powerful and well-suited for exploring early visuomotor processing in a wide range of tasks and research fields. Moreover, response priming can be dissociated from visual awareness, possibly because it is based on the first sweep of feedforward processing of primes and targets. This makes it a theoretically interesting device for separating conscious and unconscious vision. We discuss the major opportunities of the paradigm and give specific recommendations (e.g., tracing the time-course of priming in parametric experiments). Also, we point out typical confounds, design flaws, and data processing artifacts.

## An invitation to response priming

Even in a creative and prolific field like cognitive psychology, new paradigms are rare. Some are hailed as new but then recognized as variants of older ones, and only a few stand the test of time. Ideally, a new experimental paradigm allows researchers to investigate new phenomena not covered by previous paradigms, to address old questions from a different angle, and to apply new methods and insights to other domains. In vision science, one such paradigm is *response priming* (Klotz & Neumann, 1999; Klotz & Wolff, 1995; Vorberg, Mattler, Heinecke, Schmidt, & Schwarzbach, 2003). Emerging from the classical field of masked priming and unconscious perception, response priming has matured into a paradigm that can be used to investigate visual motor control, visual attention, and a hitherto unrecognized set of dissociations between rapid motor activation and visual awareness. In this paper, we will argue that response priming is a powerful and innovative tool for investigating issues of early information processing in a broad range of different fields (Schmidt et al., 2011).

**Figure 1.**
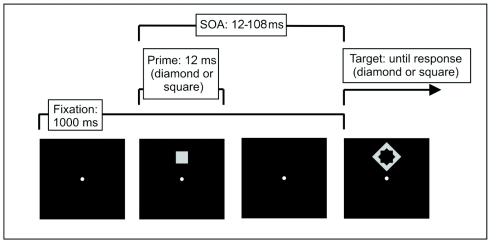
Trial in a typical response priming experiment. Primes and targets are squares or diamonds. Participants respond as quickly and accurately as possible to the contour of the target (e.g., square – right button, diamond – left button). Primes and targets appear at the same position either below or above the fixation point and can have the same shape (consistent trial) or different shapes (inconsistent trial, shown here). Primes are always presented for 12 ms, only the time interval between prime and target presentation is systematically varied. Note that targets serve as metacontrast masks for the primes.

**Figure 2.**
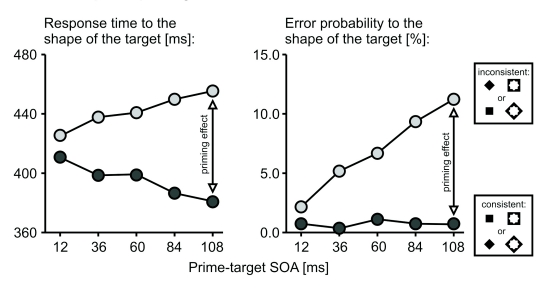
Typical time course of response priming effects (fictitious data). Consistent primes (dark gray) accelerate response times, inconsistent primes (light gray) decelerate them. At the same time, consistent primes only rarely lead to response errors, while error rates can be very high in inconsistent trials. For both response times and error rates, priming effects (differences between consistent and inconsistent trials) typically increase with SOA despite visual masking by the targets.

*Response priming* refers to a situation in which a participant is reacting to a target stimulus as quickly and accurately as possible. The target is preceded by a prime stimulus which is either mapped to the same response as the target (*consistent prime*) or to the alternative response (*inconsistent prime*, see Figure 1).^1^ Typically, consistent primes will speed and inconsistent primes will slow responses to the target, leading to *priming effects* usually defined as the response time difference between consistent and inconsistent trials. If prime and target presentations follow each other at stimulus-onset asynchronies (SOAs) shorter than about 100 ms, the resulting *priming function* (the priming effect as a function of prime-target SOA) follows a prototypical pattern where priming effects increase approximately linearly with SOA (Figure 2, left panel; cf. Vorberg et al., 2003).

Generally, response priming effects occur because the prime activates the response assigned to it. This has been shown early on in the time course of lateralized readiness potentials. These represent relative increases in EEG negativity over the motor cortices prior to response execution. They are stronger over the motor cortex contralateral to the responding hand, and hence the difference potential between the left and right hemispheres can be used as a measure of the selective preparation of a right-hand or left-hand response. Typically, the potentials start out time-locked to the prime, first develop in the direction specified by the prime, and only later proceed in the direction specified by the actual target (Eimer & Schlaghecken, 1998; Klotz, Heumann, Ansorge, & Neumann, 2007; Leuthold & Kopp, 1998; Vath & Schmidt, 2007). Such data suggest that the prime activates a response assigned to it, and has more time to drive the response on its own the more time elapses between prime and target. Therefore, priming effects increase with prime-target SOA (for a mathematical model, see Vorberg et al., 2003). Similarly, priming effects in pointing responses make it possible to trace the prime’s motor impact in the temporal as well as the spatial domain. Experiments that measured pointing movements show that inconsistent primes are not only able to delay the responses, but also to mislead them in the wrong direction. This diverting influence can lead to an initial finger movement towards the prime that only later reverses in direction of the correct target position (Schmidt, 2002; Schmidt, Niehaus, & Nagel, 2006; Schmidt & Schmidt, 2009). In many cases, response activation by an inconsistent prime can result in a full-fledged movement towards the prime position (i.e., a response error). This results in a characteristic pattern of error probabilities (cf. Figure 2, right panel). Therefore, error rates are of interest for at least two reasons. Firstly, they indicate response activation by the prime, just like the response time effects. Secondly, it is reasonable to assume that errors in inconsistent conditions are predominantly driven by prime information (e.g., Schmidt et al., 2006; Schmidt & Schmidt, 2010a). As a result, priming effects in error rates are of similar importance as the response time effects and should be analyzed just as carefully. In sum, it is the close connection between priming effects and online motor control that distinguishes response priming from other types of priming effects.^2^

A fascinating feature of response priming is its independence of visual awareness. The relationship between both can be investigated by combining response priming with methods of visual masking: If primes are rendered invisible by presentation of another stimulus in close temporal proximity (the *mask*), priming effects typically remain unchanged. By using *metacontrast masking* (Breitmeyer & Ömen, 2006), a form of masking that we will describe later, response time effects and visual awareness can even be double-dissociated: When the visibility of the primes decreases, priming effects can still increase (Albrecht, Klapötke, & Mattler, 2010; Mattler, 2003b; Vorberg et al., 2003). Thus, for a short time and under suitable experimental conditions, visually masked stimuli can influence motor responses just as effectively as clearly perceivable stimuli. This independence of visual awareness and motor activation makes response priming a particularly useful tool in studying early visual processing.

How can this independence be explained? Lamme and Roelfsema (2000; see also Lamme, 2010) propose that a novel stimulus elicits a wave of neuronal feedforward activation (*feedforward sweep*) rushing through the visuomotor system: Each cell passes activation on to cells downstream before integrating any feedback or recurrent information from other cells about the signal (also see Bullier, 2001; Thorpe, Fize, & Marlot, 1996; VanRullen & Koch, 2003). Consequently, the wavefront of visually elicited activation is essentially devoid of information from recurrent processing, which develops only in the wake of the wave. The authors suppose that such a feedforward sweep could lead to various sorts of priming processes, but not to visual awareness of the critical stimulus, which is assumed by many authors to depend on recurrent processing (e.g., Dehaene & Naccache, 2001; DiLollo, Enns, & Rensink, 2000; Lamme & Roelfsema, 2000). Applied to response priming, this theory can explain why response priming effects remain intact under visual backward masking of the prime: Because backward masking works through disruption of recurrent processing (cf. DiLollo, Enns, & Rensink, 2000; Fahrenfort, Scholte, & Lamme, 2007; Lamme, Zipser, & Spekreijse, 2002), it does not affect response priming effects resulting from feedforward activation. Evidence that response priming is based on feedforward processes comes from the previously mentioned studies of primed pointing movements and lateralized readiness potentials. These studies show that the earliest processes of response activation are determined exclusively by prime information but are independent of all target information, and that only later processed of response activation are influenced by the actual target (Klotz et al., 2007; Schmidt et al., 2006; Schmidt & Schmidt, 2009; Vath & Schmidt, 2007). Therefore, prime and target control the response in strict sequence, just as expected of a simple feedforward system.

Response priming effects are among the numerically largest effects in response time research; they can account for 25% or more of the overall response time. Therefore, the method is well suited for extensive variation of task set and stimuli. For example, it has been applied to investigate such different research areas as chess expertise (Kiesel, Kunde, Pohl, Berner, & Hoffmann, 2009) or the processing of natural images (Schmidt & Schmidt, 2009). Moreover, priming effects are not only found in keypress responses, in speeded finger pointing (e.g., Schmidt, 2002), and in electroencephalographic meaures (Eimer & Schlaghecken, 1998; Jaśkowski, Biauska, Tomanek, & Verleger, 2008; Klotz et al., 2007; Leuthold & Kopp, 1998; Vath & Schmidt, 2007), but also in speech responses (Ansorge, Klotz, & Neumann, 1998) and in eye movements (Schwarzbach & Vorberg, 2006), which can all be used to trace the time-course of motor activation by primes and targets. In the same vein, response priming effects can be assessed by brain imaging methods like functional magnetic resonance imaging (fMRI; cf. Dehaene et al., 1998; Naccache & Dehaene, 2001).

Our intention here is to give an introduction to methodological issues involved in response priming. We will discuss the most significant modulating variables and give some guidelines about parametric experimentation. Our goal is to ensure that researchers new to the paradigm can make full use of its temporal dynamics and avoid important confounds. We will also consider some potential pitfalls and possible misconceptions that may help avoid some of the less obvious mistakes in setting up experiments and analyzing the data. In the end, everything will be condensed into a non-comprehensive list of “Dos and Don’ts” which we hope will be helpful to newcomers to the field. It shouldn’t be observed too dogmatically: Even though it is wise to avoid certain mistakes and artifacts, in some situations it will be of theoretical interest to systematically go against those recommendations.

## Utilizing the time-courses of masking and priming

### The power of parametric variations

Usually, response priming effects (in response times and error rates) are numerically large compared to other effects in response time research, and pretty stable across individuals, so that reliable results can be obtained from comparatively small samples. This makes response priming well suited for parametric variation of the independent va-riables, and in fact the paradigm unfolds its true power only in parametric experiments.

Here, we would like to argue for a more frequent use of parametric experiments in response priming research but also in cognitive science in general. Firstly, parametric experiments allow for the examination of a dynamical range of effects. If a researcher is not sampling enough levels of an independent variable or levels that are too similar, the true functional relationship with the dependent variable may be overlooked. For example, dose-response studies in medicine can be used to determine how much of some medication is needed to have a sufficient effect. Similarly, knowing how response priming effects increase with prime-target SOA will help find suitable conditions for obtaining large effects. Secondly, only parametric variation can reveal unexpected nonlinearities (e.g., a change of sign in the dependent variable). This is of special importance when the effect of interest is not well known yet. For example, not any amount of some medicine is normally yielding positive effects, but may act benevolently or malevolently depending on the actual dose (similarly, as discussed below, response priming effects can be qualitatively different for different ranges of prime-target SOA). Thirdly, parametric variation naturally achieves an internal cross-validation of an effect. Even if the effect is strongly modulated across experimental conditions, its basic pattern normally repeats at most levels of the independent variable, allowing for an evaluation of the reliability of an effect within a single experiment.^3^

### The time-course of response priming

The most prominent and influential variable in response priming experiments is the SOA, that is, the time interval between prime and target onset (tightly linked to the *interstimulus interval*, ISI, which is defined as the time between the offset of the prime and the onset of the target – consequently, it equals SOA minus prime duration). We have already seen that response priming effects strongly increase with SOA: The longer the prime signal can control the motor response before the target signal does, the stronger the prime’s influence on the output variables (Figure 2)). This way, even very shortly presented primes can exert a large influence on a subsequent response, but only as a function of the time that is available before the target captures motor control (Vorberg et al., 2003).

Varying the SOA offers the opportunity to study the time course of rapid visuomotor processing in detail. Different sorts of stimuli and different phenomena of visual processing (e.g., visual attention, perceptual grouping, stimulus contrast) may be characterized by their specific influence on the time course of response priming. For example, if participants are responding to primes and targets that are based on perceptual grouping, strong perceptual grouping allows for a steeper priming function than weaker grouping. This way, different grouping principles can be compared with respect to their visuomotor processing dynamics (Schmidt & Schmidt, 2010b). Similarly, visual attention to locations or features can steepen the slope of the priming function (Schmidt & Schmidt, 2010a; Schmidt & Seydell, 2008), just like low-level stimulus variables such as color saturation do (Schmidt et al., 2006). Without variation of the SOA, crucial information about the slope of the priming function is missed.^4^

Knowing the entire priming function is crucial for understanding nonlinearities in the priming effect. A case in point is what can happen if three stimuli are employed (prime, mask, and target) and the prime-target SOA exceeds 100 ms. For these long SOAs, the size of the priming effect may further increase, but, under specific conditions, may also switch sign (so that participants are responding faster in inconsistent trials than in consistent trials). This reverse priming is often called the *negative compatibility effect* (Eimer & Schlaghecken, 1998, 2003). The exact conditions under which this reversal occurs are still debated, and several theories compete to explain the effect (see Sumner, 2007, for a review). If nonlinearities such as the negative compatibility effect are not taken into account, comparisons of priming effects at only a single SOA level can be wildly misleading. For instance, if a priming effect in condition A is positive while the effect in condition B is negative, this can either be due to qualitative processing differences between the conditions or to the fact that a positive-to-negative time-course is present in both conditions but captured in different states of development (Lingnau & Vorberg, 2005). Sampling entire priming functions in both conditions would resolve the ambiguity.

The systematic variation of SOA is inevitably accompanied by a large number of experimental conditions, at least compared to stan-dard experiments in cognitive science. Nevertheless, we believe that it is absolutely necessary to thoroughly understand the time course of any effect studied, and to compare entire priming functions from different conditions. Our recommendation is that in every response priming experiment, the SOA should be varied in at least three steps, covering the whole time range of interest (e.g., from 0 to 100 ms). To produce reliable data patterns with relatively small standard errors, around 60 trials per condition and participant has turned out to be a reasonable number that also makes it possible to evaluate entire data patterns in single participants.

### Double-dissociated time-courses of priming and awareness

As argued above, an outstanding feature of response priming is its capability to explore dissociations between rapid motor activation by the primes and visual awareness of them. This can be achieved by contrasting response priming effects or other indicators of processing speed with measures where participants report the final outcome of processing in visual awareness. The most common method to syste-matically control prime visibility is to present a masking stimulus either at the same time (*simultaneous masking*), shortly before (*forward masking*), or after prime presentation (*backward masking*; see Breitmeyer & Ömen, 2006). This can be achieved either by letting the target itself act as a masking stimulus for the prime (two-stimulus sequence of prime and mask/target) or by introducing the mask as an additional stimulus (three-stimulus sequence of prime, mask, and target). A type of masking often used in response priming studies is *metacontrast*, a form of backward masking in which the inner contours of a hollow masking stimulus are bordering the outer contours of the previously presented prime (cf. Figure 1)). The amount of masking (or prime visibility) is measured by administration of a *prime identification task*, in which the participant is asked to detect, identify, or categorize the prime, or to indicate its subjective visibility. While the prime identification task is considered a direct measure of visual awareness of the prime, the priming effect can be viewed as an indirect measure of prime processing (Reingold & Merikle, 1988).

**Figure 3.**
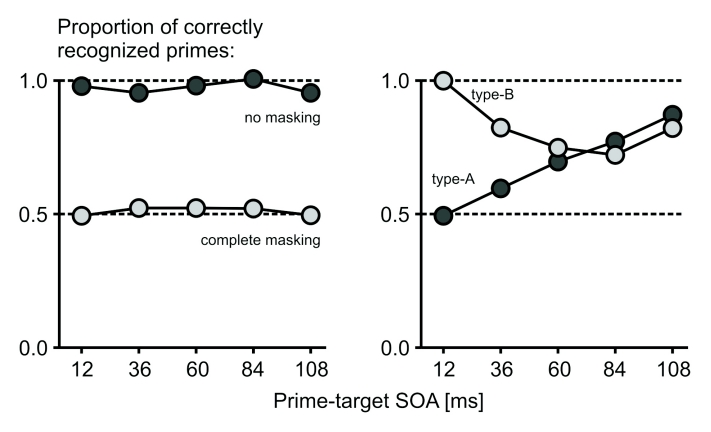
Typical patterns of backward masking (fictitious data). The proportion of correctly identified primes depends on the strength of masking. Without masking, the prime is correctly identified in virtually 100% of trials (dark gray, left panel), while identification performance at chance level (50%, light gray, left panel) would indicate complete masking. Depending on mask type, other time courses can be achieved (right panel). Under type-A masking, masking is strongest when prime and target follow each other in rapid succession and becomes weaker with increasing SOSOA (dark gray, right panel). In type-B masking, masking is strong at intermediate SOSOAs around 50 ms and weaker at shorter or longer SOSOAs (light gray, left panel). Type-B masking occurs under specific stimulus conditions in metacontrast masking.

In general, different kinds of masks lead to different *masking functions*, which describe masking as a function of prime-mask SOA (Figure 3). The most prominent masking functions are type-A and type-B masking. In *type-A masking*, prime visibility is lowest at short prime-mask SOAs and increases with increasing SOA (Figure 3, right panel). While type-A masking is the most commonly observed masking function, *type-B masking* occurs under specific circumstances in metacontrast masking. Here, visibility is lowest at medium SOAs (often, around 50 ms) and higher at shorter or longer SOAs (Figure 3, right panel). In both type-A and type-B masking, the strength and time-course of masking depend on a number of factors, including stimulus attributes of primes and masks (e.g., energy and saturation), prime-mask SOA, crowding by irrelevant distractor stimuli, and marked individual differences (Albrecht et al., 2010; Breitmeyer & Ömen, 2006; Schmidt & Schmidt, 2010a). As a result, it is possible to produce a variety of qualitatively different masking functions if parameters are carefully adjusted.

As argued above, response priming and masking can have qualitatively different time-courses, which can be assessed by varying the prime-target SOA and by comparing the resulting priming and masking functions. Interestingly, the time-courses of priming and prime visibility can form a double dissociation where both variables develop in opposite directions (Schmidt & Vorberg, 2006; also see Merikle & Joordens, 1997a, 1997b). For instance, if response priming is combined with type-B masking, there will be a range of prime-mask SOAs that lead to decreasing visibility of the prime yet increasing priming effects. Under very mild measurement assumptions, double dissociations imply that priming and awareness cannot both be driven by a single source of conscious information, thus refuting the idea that priming effects may simply measure residual conscious information about the prime not detected by the prime identification task (see Schmidt & Vorberg, 2006, for mathematical proofs).

In this way, double-dissociations overcome the problem of the traditional *zero sensitivity criterion*, which demands that priming effects must be demonstrated under conditions where sensitivity to the prime is exactly zero (i.e., when the prime is “invisible”). This criterion is seemingly strict but implies strong measurement assumptions which are not required for double dissociations (most critically, it requires an exhaustive measure of visual awareness which is guaranteed to never miss a change in visual awareness, however tiny; cf. Reingold & Merikle, 1988). In fact, the common preconception of many researchers (and many reviewers) that masked priming experiments require zero sensitivity for the prime is a fallacy, because double dissociations are invariably more informative, more powerful, and less assumption-ridden.^5^

 Importantly, double dissociations are not confined to metacontrast masking, or even to masking in general. For example, Schmidt et al. (2010) reported a double dissociation between priming effects and the subjective appearance of the prime in a visual brightness illusion, showing that under specific circumstances one prime may look brighter than the other but prime responses as if it was darker (and vice versa). This dissociation was achieved without any masking, with all stimuli clearly visible.

## Things that can go wrong

Response priming is a powerful method with numerous applications. However, there are pitfalls that should be avoided when applying the method.

### Degrading the prime signal

During most of the history of unconscious perception, the zero-sensitivity criterion seemed to be the only way to investigate perception without awareness. In order to decrease the visibility of the prime, many researchers resorted to decreasing its stimulus energy (e.g., intensity, contrast, color saturation, or duration) until prime identification performance fell below some strict threshold. However, degrading the prime signal means diminishing the priming effect: Since response priming increases with increasing prime energy (e.g., Schmidt et al., 2006), any strong reduction in prime signal strength will abolish the priming effect. In other words, in any design which modifies visibility by varying the prime, the indirect measures (e.g., priming task) and direct measures (e.g., prime identification task) are always confounded. As a result, priming effects will increase along with the visibility of the prime, erroneously suggesting a strong relationship between response priming and visual awareness.

Even more havoc comes from varying the prime’s duration while leaving the prime-target ISI constant. This, of course, confounds prime duration and prime-target SOA and creates a spurious correlation between priming and prime visibility by two mechanisms: firstly, by confounding prime visibility with prime energy (because stronger prime implies stronger priming), and secondly, by confounding it with SOA (because longer SOA implies stronger priming).

Unless variation of the prime is of theoretical interest, it is therefore crucial to leave the prime intact and control its visibility by variation of mask attributes only (e.g., its stimulus contrast). With higher mask energy, visual awareness for the prime should diminish (Breitmeyer & Ömen, 2006). This way, visual awareness can be varied indepen-dently of prime-mask SOA. We recommend varying prime visibility in several steps (at least two) to enable detection of a double dissociation.

### Avoiding D-I mismatch

 Any type of dissociation between direct (D) and indirect measures (I) depends on a direct comparison of two tasks. Therefore, mismatch of direct and indirect tasks should be avoided. Following the recommendations by Schmidt and Vorberg (2006) , we strongly recommend that direct and indirect tasks be matched with respect to (a) the stimuli, (b) the features to be judged, and (c) the assignment of those features to motor responses. Ideally, tasks should only differ in whether the prime or target is the imperative stimulus. 

 For example, Schmidt and Schmidt (2009) employed a target identification task where participants had to decide as quickly as possible which of two simultaneously presented target pictures contained an animal. Target pictures were preceded by one animal and one non-animal picture at the same positions as the targets. A matched direct task would adopt exactly the same experimental setup but would ask for a decision which of the two prime pictures showed an animal. Stimuli and stimulus-response mappings would be identical in both tasks. Crucially, the direct task would assess precisely the information driving the priming effect in the indirect task, namely which of the two pictures contained the animal. An example of D-I mismatch would be to ask participants to freely report prime picture contents instead of performing a forced choice between animal and non-animal pictures. Other examples would be to present only one prime and ask participants whether or not it contained an animal, or to ask them if a prime or no prime was presented (both are detection tasks). In all such cases, the direct task loses validity: It becomes impossible to tell what performance in the direct task has to say about visual awareness for the primes in the indirect task because the type of information driving performance in both tasks is not the same. 

 In some studies, participants are asked to perform direct and indirect tasks in the same trial, for instance, a speeded forced-choice target discrimination followed by forced-choice prime discrimination. This procedure has the advantage of allowing for trial-by-trial correlations between measures. The disadvantage of this method is that it creates a dual-task situation where performance in either task may suffer. Dual-task tradeoffs may also be viewed as a source of D-I mismatch where the mismatch arises from uneven splits of attention between both tasks. Regarding indirect measures, results from Vorberg et al. (2003) suggest that priming effects are not affected by a subsequent (unspeeded) prime identification judgment. Regarding direct measures, however, syste-matic comparisons between the single-task and dual-task approaches are still missing. Therefore, we generally recommend administering direct and indirect tasks in separate blocks to ensure optimal attention to the relevant stimuli.^6^

In principle, visual awareness of a prime can be measured either by subjective or objective methods, depending on the research question of interest (Cheesman & Merikle, 1984). For example, participants can rate the confidence with which they were able to identify the prime (subjectively), or they can perform a forced-choice discrimination between two possible spatial positions of the relevant prime (objectively). One type of measure cannot replace the other, and both give potentially interesting information on visual awareness. However, it is often difficult to match subjective measures to the indirect measure. Therefore, we recommend using subjective measures only in tandem with objective measures.

### Strategic confounds

In general, it is advisable to intermix the different experimental conditions in response priming experiments. An important exception are studies where a large range of prime-target SOAs is studied. When all SOAs are randomly intermixed and the proportion of long SOAs is high, participants will be forced to adjust their response criteria to those long SOAs to avoid response errors. As a result, their responses will be delayed, and the hallmark properties of response priming may not be obtained. The same pattern can be observed when in some of the trials participants must not respond at all (*nogo condition*; see Klotz et al., 2007). We advise to block short SOAs (say, up to 100 ms) and longer SOAs (longer than 100 ms) to avoid such confounds.

### Dealing with motor properties of the masks

With regard to response priming, it is either possible to let the target act as a masking stimulus for the prime (two-stimulus sequence of prime and mask/target, see Figure 1) or to introduce the mask as an additional stimulus (three-stimulus sequence of prime, mask, and target). The former approach requires that the particular target stimulus is adequate for masking the prime. Of course, it inevitably confounds the SOA between prime and mask (which defines the masking function), with the SOA between prime and target (which defines the priming function); therefore, it might be desirable to vary prime-mask SOA and prime-target SOA independently. However, the time-course of motor activation in the three-stimulus situation is not well understood, and intervening masks seem to be able to interact with primes and targets on a motor level. For example, response-relevant masks (masks that consist of a superposition of response-relevant prime features) have been shown to strongly affect negative compatibility effects (Jaśkowski, 2008; Jaśkowski et al., 2008; Verleger, Jaśkowski, Aydemir, van der Lubbe, & Groen, 2004). If influences of the mask on the priming effect are to be avoided, masks should not be response-relevant themselves, that is, they should not possess properties of the prime or target stimuli that would potentially induce a motor response by themselves. Unwanted motor effects from the mask seem to be especially large if the mask closely follows the prime, that is, occurs early in the prime-target interval.^7^

### Spatial confounds

 A major advantage of the response priming paradigm is the possible variety in number and spatial arrangement of the stimuli employed. Specifically, one or several prime and target stimuli may either be presented at the same location or at different locations (e.g., the prime below and the target above fixation, two primes flanked by two targets, two targets flanked by two primes, etc.). For instance, Schmidt and Seydell (2008) and Schmidt and Schmidt (2010a) employed a paradigm in which 10 primes were followed by 10 targets at the same positions, and Schmidt and Schmidt (2010b) presented two primes that were subsequently flanked on the left and right by two targets. Although number and spatial arrangement of stimuli notably differed, the basic patterns of results were typical for those of other response priming experiments.^8^

 However, care should be taken to avoid confounding response priming effects with spatial artifacts. In most situations, it is desirable that spatial compatibility effects be avoided (e.g., by not presenting primes and targets to the left and right of fixation when key responses are also left and right, by counterbalancing all experimental conditions across sessions and participants, and by randomizing all stimulus positions). A strategy especially suitable for avoiding spatial artifacts is to let participants choose between two targets preceded by two primes, with prime positions either spatially consistent or inconsis-tent (switched) with respect to target positions. For example, Schmidt and Schmidt (2009) asked participants to decide which of two target images contained an animal, with an animal and a non-animal prime presented at the same two locations. Compared to the single-prime-single-target situation, this procedure turns a yes-no decision task into a two-alternative forced-choice task (Macmillan & Creelman, 2005) where the response is always directed by the spatial position of the relevant target, and spatial compatibility effects are neutralized.

It has been shown that spatial eccentricity alters both the magnitude and the time-course of response priming effects (Lingnau & Vorberg, 2005). Therefore, eye movements should be controlled, and participants should be instructed to keep fixation during the entire trial. In our opinion, it is not strictly necessary to use an eye-tracking device to control for eye movements:

A sufficient technique is to set up the stimuli in such a way that their possible positions are arranged symmetrically around the fixation point. This way, participants are discouraged from using fixation stra-tegies because the best strategy is to always look at the fixation point. Also, results from spatial pre-cueing tasks suggest that participants are capable of maintaining steady fixation with very little eye movements if explicitly asked to (Abrams & Law, 2000; Arrington, Carr, Mayer, & Rao, 2000; Thiel, Zilles, & Fink, 2004; Yantis & Jonides, 1990). Finally, in many response priming experiments, time intervals between primes and targets are too brief to permit eye movements anyway.

### Artifacts in measuring prime visibility

Recently, some researchers have adopted the practice of evaluating prime visibility separately for each participant and then discarding those participants who perform significantly above chance (or meet a similar criterion). The implicit assumption is that for the remaining participants, the critical stimulus must be invisible. A variant of this practice is to look at a range of confidence judgments of a single participant and then to eliminate all trials which indicate some degree of visibility, concluding that stimuli in the remaining trials must be invisible.

This method suffers both from conceptual and from measurement-theoretical problems. On the conceptual level, it is guilty of a sampling fallacy: Discarding some observations from the sample does not change the underlying population on which the sample is based. Actually, the practice is analogous to eliminating all the patients from a clinical trial that have died from the drug under investigation. On the measurement level, the approach is taking observers’ judgments that an observation belonged to the lowest visibility category (labeled “unaware”) at face value: It is simply assuming that the visibility ratings truthfully reflect the actual subjective visibility. This would require two assumptions: firstly, that there indeed are internal states of “awareness” and “unawareness”, and secondly, that the participants are perfectly able to classify those internal states. In reality, however, there will be a continuum of internal states, and participants will need to impose decision criteria (with unknown response biases) to classify them into ratings. Under such a scenario, each rating category must be expected to contain misses and false alarms from adjacent categories, and it is implausible to assume that the rating procedure will identify a set of truthfully “unconscious” trials. Basically, the procedure ignores the modern psychophysical view that a participant’s response behavior is determined jointly by the subjective visibility of the stimulus, by the set of response criteria forming the decision boundaries of the different response categories, and by chance factors (Green & Swets, 1966; Macmillan & Creelman, 2005).

Sometimes, the question arises whether masking functions should be analyzed separately for consistent and inconsistent trials (this is often suggested by reviewers). Again, the basic problem with this approach becomes obvious when judged from the perspective of signal detection theory: Calculating the sensitivity measure *d’* separately for consistent and inconsistent trials confounds sensitivity and bias. Vorberg, Mattler, Heinecke, Schmidt, and Schwarzbach (2004) show that for the standard signal-detection model assuming identical normal distributions, *d’*_con_ = *d’* + Δβ while *d’*_incon_ = *d’* - Δβ, where Δβ = *z*(*F*_Target 1_) - *z*(*F*_Target 2_) is the differential effect (in *z* units) of the targets on the false alarm rates in each target condition. Similar problems will occur with percent-correct and other sensitivity measures. The way to avoid such sensitivity-bias confounds is to follow the stan-dard recommendation from signal detection theory: Keep conditions constant that could affect response bias. One way of doing this is to calculate *d’* separately not for different consistency conditions, but for different target types (Vorberg et al., 2003, 2004). 

However, plotting masking functions separately for consistent and inconsistent trials can reveal interesting response strategies employed by single participants. Such plots typically exhibit strong interactions between prime-target consistency and prime-target SOA: Typically, identification performance at short SOAs is very high for consistent primes and very low (often below chance) for inconsistent primes, while the opposite may occur at longer SOAs. When participants are asked about their strategies, they frequently report guessing the prime by the perceived amount of flicker in the prime-target pair. If flicker is weak, observers assume that the prime is consistent with the target and respond accordingly; when flicker is strong, they conclude that the prime is inconsistent and give the opposite response. However, the amount of flicker is in fact driven primarily by the prime-target SOA, with more flicker at longer SOAs. As a result, responses reveal a strong bias in target direction at short SOAs, but a bias against target direction at long SOAs (for exemplary data and discussion of this bias, see Schmidt, 2000).

Similarly, in metacontrast masking participants may try to identify consistent and inconsistent trials by judging the amount of apparent motion between masked primes and visible targets (i.e., rotation). However, even while participants are able to detect this motion cue in a separate task, this performance has been shown to correspond neither with the participants’ prime identification performance nor with the resulting priming effects (Ansorge, Becker, & Breitmeyer, 2009; Ansorge, Breitmeyer, & Becker, 2007).

## Grand designs

Response priming is a powerful paradigm that can be employed to examine the time-course of visuomotor processing in depth. In our opinion, the basic mechanisms of response priming are now reasonably well understood and have led to detailed and successful theories (e.g., the principle of direct parameter specification by Neumann, 1990; the action trigger account by Kunde, Kiesel, & Hoffmann, 2003; and the rapid-chase theory by Schmidt et al., 2006). Implementation is fairly easy as long as researchers avoid the pitfalls discussed in the present paper (see Box 1). Because of its dissociability from visual awareness, response priming is especially suited for studying the effects of prime stimuli on preconscious processing, and possibly processing based primarily on a first feedforward sweep of visuomotor information processing. The method allows for a wide range of visual stimuli, decision tasks, dependent variables, and spatial arrangements, and therefore has high potential to be applied to a wide spectrum of issues in cognitive science. Because response priming effects are typically large, the method lends itself to parametric experimentation.

**Box 1.** Dos and Don’ts in Response Priming Research.

DO	DON’T
Vary prime-target SOA in at least three steps to know the time-course of priming.	Create D-I mismatch.
Compare priming effects by comparing entire priming functions.	Masked priming: Vary visibility by changing the prime insteadof the mask.
Aim at parametric variation of other variables.	Confound prime presentation time and SOA.
Analyze response times as well as error rates.	Mix very long and very short SOAs.
Employ online measures of motor activation.	Use visibility ratings to classify trials or subjects as „unaware”.
Look for double dissociations between priming and awareness.	Believe that the zero-awareness criterion is the only way to go.
Masked priming: Be careful with motorically active and early masks.	

In our lab, we have just begun to realize some of the many possible applications of the response priming paradigm. So far, we used response priming as a method to investigate visual awareness and online motor control (e.g., Schmidt, 2002), visual attention (Schmidt & Schmidt, 2010a; Schmidt & Seydell, 2008), brightness processing (Schmidt et al., 2010), and natural image processing (Schmidt & Schmidt, 2009). Currently, we are further extending our research interests by using the response priming method to investigate such different topics as visual processing of transparency, processing advantages of phobic stimuli, illusory contours, figure-ground segregation, and perceptual grouping (see Schmidt et al., 2011, for some examples). Other labs used the method to investigate unconscious color processing (Breitmeyer, Ro, & Singhal, 2004), unconscious number processing (Dehaene et al., 1998), priming of cognitive control operations (Mattler, 2003b), or the role of expertise in unconscious processing in chess players (Kiesel et al., 2009) or typists (Heinemann, Kiesel, Pohl, & Kunde, 2010).

Accordingly, we think of response priming not primarily as an interesting effect in visuomotor processing and cognition, but as an experimental method that is heavily underestimated and underemployed considering its scientific potential. The basic properties of response priming sequential response activation by primes and targets, independence of priming from awareness, and possibilities for variation raise the possibility of contrasting the results of early processing (preconscious and presumably based on a feedforward sweep) with those of late processing (conscious and based on recurrent processing) for many phenomena in human visual cognition (Schmidt et al., 2011). Response priming methods can thus complement or even be infused into studies in neurophysiology and neuroimaging, especially regarding the potential for double dissociations between priming and visual awareness.

## Footnotes

1 While we prefer the term *consistency* to refer to the prime-target relationship, the terms *congruency* and *compatibility* are also in use. Alternative names of the response priming paradigm itself are *action priming*, *target priming*, and *metacontrast dissociation*.

2 In particular, response priming can be clearly distinguished from semantic priming because it does not require a semantic connection between prime and target. While most semantic priming effects are probably unrelated to response priming, some types of semantic classification might be able to activate fast motor responses to produce a semantic response priming effect. Possible examples include the decision whether a single-digit number is smaller or larger than 5 (Dehaene et al., 1998), decisions which of two target pictures contains an animal (Schmidt & Schmidt, 2009), or even chess-players’ decisions whether or not the king is in check (Kiesel, Kunde, Pohl, Berner, & Hoffmann, 2009). We will not discuss semantic priming here but refer to reviews by Lucas (2000) , by Hutchison (2003) , and by van den Bussche, van den Noortgate, and Reynvoet (2009).

3 We believe that parametric methods are underused specifically in neuroimaging studies, for example, regarding the technique of correlating changes in the blood oxygenation signal to different levels of a quantitative independent variable.

4 Note that these modulatory effects of attention do not imply that priming is based on cognitive control processes. Instead, visual attention modulates the priming effect if directed to features or locations just in time before prime onset, boosting feedforward processing of primes and targets in the upcoming trial (Schmidt & Schmidt, 2010a; Schmidt & Seydell, 2008). A similar argument applies to studies showing the mediation of response priming effects by intentional response strategies (e.g., Kunde, 2003): Here, top-down control might adjust response thresholds in upcoming trials.

5 Specifically, let *I_i_*(*c_i_*, *u_i_*) and *D_i_*(*c_i_*, *u_i_*) be two measures of visual processing in experimental condition *i*, one indirect (e.g., priming effect) and one direct (e.g., prime identification performance), such that both measures are functions of conscious (*c*) as well as unconscious (*u*) sources of visual information. A double dissociation is observed when for two experimental conditions *i* and *j*, *I_i_*(*c_i_*, *u_i_*) > *I_j_*(*c_j_*, *u_j_*) while *D_i_*(*c_i_*, *u_i_*) < *D_j_*(*c_j_*, *u_j_*), or vice versa. Schmidt and Vorberg (2006) show that this data pattern implies nonzero unconscious information (*u* > 0) in at least one condition. Only assumptions of weak monotonicity with respect to conscious information need to be made for *D* and *I*, while the traditional zero-sensitivity criterion (which demands that *D* = 0) requires unrealistically strong assumptions of strict monotonicity.

6 Unfortunately, trial-to-trial correlations between direct and indirect measures are often of limited use. *Zero correlations* in a single condition do not imply zero correlation across conditions, which are typically of major interest when prime visibility is varied experimentally. *Positive correlations* may be due to third variables (e.g., fluctuations in alertness) and do not allow for assessment of the causal direction of the relationship; also, their maximal size is severely restricted by measurement noise. *Negative correlations* would be interesting but have never been reported.

7 Unpublished data from our lab suggest that early masks immediately following the prime can also impede regular response priming effects.

8 The classical flanker paradigm by Eriksen and Eriksen (1974) might be a special case of the response priming paradigm: Both techniques seem to yield similar results when the same time range is studied (cf. Mattler, 2003a; Schwarz & Mecklinger, 1995; Vorberg et al., 2003). Even in their seminal 1974 paper, Eriksen and Eriksen showed that response times were the same for flankers identical to the targets and for nonidentical flankers assigned to the same response as the target, thus demonstrating flanker effects on a motor rather than on a perceptual level.
